# Unveiling the gut microbiota blueprint of schizophrenia: a multilevel omics approach

**DOI:** 10.3389/fpsyt.2024.1452604

**Published:** 2024-09-25

**Authors:** DongDong Qi, Peng Liu, YiMeng Wang, XuGuang Tai, ShiFa Ma

**Affiliations:** ^1^ Basic and Clinical Laboratory of Mental Illness, Hulunbuir Third People’s Hospital (Hulunbuir Mental Health Center), Yakeshi, Inner Mongolia, China; ^2^ School of Public Health, Inner Mongolia Medical University, Hohhot, Inner Mongolia, China

**Keywords:** schizophrenia, metabolomics, 16S rRNA gene sequencing, enrichment pathway, co-expression analysis

## Abstract

**Background:**

Schizophrenia is a persistent incurable mental disorder and is characterized by the manifestation of negative emotions and behaviors with anxiety and depression, fear and insecurity, self-harm and social withdrawal. The intricate molecular mechanisms underlying this phenomenon remain largely elusive. Accumulating evidence points towards the gut microbiota exerting an influence on brain function via the gut-brain axis, potentially contributing to the development of schizophrenia. Therefore, the objective of this study is to delineate the gut microbial composition and metabolic profile of fecal samples from individuals with schizophrenia.

**Methods:**

Liquid chromatography-mass spectrometry (LC-MS) and 16S ribosomal RNA (16S rRNA) gene sequencing were employed to analyze fecal metabolites and gut microbiota profiles in a cohort of 29 patients diagnosed with schizophrenia and 30 normal controls. The microbial composition of fecal samples was determined through the 16S rRNA gene sequencing, and microbial α-diversity and β-diversity indices were calculated. Principal component analysis (PCA) and orthogonal partial least squares discriminant analysis (OPLS-DA) were performed to analyze the distribution of samples. The metabolites and gut microbiota exhibiting differential expression were identified through the application of biological variance criteria. Co-occurrence analysis of bacteria and metabolites was conducted using the spearman’s rank correlation coefficient and visualized in a circular layout with the Cytoscape software.

**Results:**

The findings of the study indicated a lack of substantial evidence supporting significant disparities in α-diversity and β-diversity between individuals with schizophrenia and normal controls. In terms of metabolomics, a discernible pattern in sample distribution between the two groups was observed. Our analysis has revealed 30 bacterial species and 45 fecal metabolites that exhibited notable differences in abundance between individuals diagnosed with schizophrenia and normal controls. These alterations in multilevel omics have led to the development of a co-expression network associated with schizophrenia. The perturbed microbial genes and fecal metabolites consistently demonstrated associations with amino acid and lipid metabolism, which play essential roles in regulating the central nervous system.

**Conclusion:**

Our results offered profound insights into the impact of imbalanced gut microbiota and metabolism on brain function in individuals with schizophrenia.

## 1 Introduction

Schizophrenia is a severe psychiatric disorder characterized by the presence of psychotic symptoms, alongside a range of negative symptoms, and dysfunction in reward processing, accompanied by sensory, emotional, and behavioral disturbances. It affects approximately 1% of the global population (1, 2), and contributes significantly to morbidity, resulting in causes 12.7 million Disability-Adjusted Life Years (DALYs) worldwide (3). The incidence rates of schizophrenia and autism are roughly the same (4), at approximately 1%, which is lower than that of anxiety and depression, which have an incidence rate of 4% (5, 6).

The etiology and pathology of schizophrenia are complex, involving a combination of genetic (7), neurotransmitter (8), inflammation (9), and oxidative stress factors (10), which is still one of the main causes of the low cure rate of the disease, even through three generations of anti-schizophrenia drugs have made significant updates and advances in recent years (11).

Despite extensive research, the exact cause of schizophrenia remains unclear, and diagnosing the disorder is complicated by its wide range of symptoms (12). In the field of psychiatric care, advancements have been made in the development of antipsychotic drugs to enhance treatment efficacy and minimize adverse effects. However, the cure rate for schizophrenia has yet to surpass 20% (11, 13, 14). Over half of individuals will experience a recurrence of schizophrenia during their lifetime, leading to significant impacts on their physical and mental well-being, as well as socioeconomic status (15). Currently, there is a deficiency in accessible, affordable, and widely available diagnostic tools to effectively address this global mental health challenge. The diagnosis of schizophrenia predominantly depends on clinical presentation and assessments conducted by experienced psychiatric professionals following the guidelines outlined in the Diagnostic and Statistical Manual of Mental Disorders Fifth Edition (DSM-5) (16). Therefore, it is imperative to elucidate the molecular mechanisms and novel biomarkers for the management and treatment of disease.

The human gut microbiome, considered a microbial organ, exerts significant influence on health and disease (17). Of particular interest is its role in the development of various the central nervous disorders, including major depressive disorder (18), Alzheimer ‘s disease (19), Parkinson’s disease (20), and autism (21).16S rRNA gene sequencing and shotgun metagenome sequencing are prominent technologies utilized for the identification of potential pathogens within the gut, thereby elucidating their role in disease development (22). The “microbiota-brain-gut axis” concept has garnered significant attention from researchers, unveiling a bidirectional communication pathway between the gut microbiome and the central nervous system, commonly referred to as the “microbiota-gut-brain axis” (23, 24). Studies have demonstrated the ability of gut microbiota to produce or stimulate neurotransmitters, serotonin (25), dopamine (26), and γ-aminobutyric acid (GABA) (27), which are essential for regulating mood, cognition, and overall mental health.

Metabolomics, an emerging discipline within systems biology, plays a crucial role in various fields, including disease diagnosis and other areas pertinent to human health care (28). This scientific field involves the qualitative and quantitative analysis of small molecular metabolites in organisms, elucidating the interplay between metabolites and physiological and pathological alterations resulting from environment or genetic perturbations (29). Metabolomics serves as a bridge connection for internal factors such as genes and proteins to observable phenotypic traits. Metabolomics has been utilized to explore specific biomarkers relevant to schizophrenia, such as ceramide, acetylcarnitine, and γ-aminobutyric acid, which plays an important role in maintaining neuronal excitability and maintaining the balance of the nervous system (30).

In order to fill gaps in our understanding of how gut microbiota and metabolites are altered in schizophrenia, a study was conducted on 59 fecal samples obtained from individuals with schizophrenia and normal controls. We aimed to analyze the changes in bacterial composition and fecal metabolites at multiple levels, as well as their interplay within the gut ecosystem of individuals with schizophrenia. Our objective was to elucidate the impact of these altered signatures on host metabolism.

## 2 Materials and methods

### 2.1 Subject recruitment

The research protocol received approval from the ethics committee of Hulunbuir Mental Health Center (No. 2021NO.02) and adhered to the guidelines set forth in the Declaration of Helsinki. This study included 59 participants, ranging in age from 22 and 64, comprising 29 normal controls and 30 individuals diagnosed with schizophrenia. Every individual involved in the research study gave consent in writing after being informed about the study. To be eligible for the study, patients had to adhere to the diagnostic criteria for schizophrenia, as defined in the fifth edition of the Diagnostic and Statistical Manual of Mental Disorders (DSM-V) (16). The patients in the disease group included in this study were inpatients who had received cross-treatment with ziprasidone hydrochloride, clozapine, and lorazepam to enhance drug efficacy, reduce side effects, or address refractory symptoms, moreover, the average hospital stay for these patients extends for over half a year. At the same time, the meals of enrolled patients were standard meals by the hospital.

Normal controls were recruited through an annual hospital staff examination to confirm the absence of any history of psychiatric disorder as defined by the DSM-V. Exclusion criteria encompassed (1) a lifetime history of bipolar disorder; (2) chronic inflammatory disorders, diabetes, cardiovascular disease, thyroid disease, or cancer; (3) alcohol abuse, drugs abuse, smoking history, or acute poisoning; (4) current pregnancy or breastfeeding; and (5) no diet habit or antibiotic use in the last month.

### 2.2 Fecal sample collection

Fecal samples were collected in compliance with ethical standards established by the Hulunbuir Mental Health Center, with the aid of medical professionals. Participants were given sterile fecal collector beforehand, and the central portion of the fecal matter was gathered. The collected morning fecal samples were then immediately preserved in a -80° freezer for subsequent DNA extraction.

### 2.3 16S rRNA gene sequencing analysis of fecal samples

#### 2.3.1 Genomic DNA extraction and amplification

To extract total genomic DNA samples, the OMEGA Fecal DNA Kit (catalog no. D4015-02) (OmegaBio-Tek, Norcross, GA, USA) was utilized precisely following the manufacturer’s guidelines. The OD260/OD280 ratio of all extracted DNA samples were less than 1.9, indicating a good quality of DNA samples. The average DNA concentration was 90.45 ± 20.34 ng/μL.

Quantity assessment was conducted using a NanoDrop NC2000 spectrophotometer (Thermo Fisher Scientific, Waltham, MA, USA), while quality was evaluated through agarose gel electrophoresis. The extracted DNA samples were then stored at -20° for subsequent analysis. For the amplification of the bacterial 16s rRNA genes v3-v4 region, the forward primer 338F (5’-ACTCCTACGGGAGGCAGCA-3’) and the reverse primer 806R (5’-ACTCCTACGGGAGGCAGCA-3’) were employed. Unique 7-bp barcodes were incorporated into the primers, facilitating multiplex sequencing. The PCR reaction mixture was precisely formulated, consisting of 5 μL of 5X buffer, 0.25 μL of Fast pfu DNA Polymerase (5U/μL), 2 μL of dNTPs (2.5 mM), 1 μL of each Forward and Reverse primer (10 μM), 1 μL of DNA template, and 14.75 μL of ddH2O. The thermal cycling conditions were carefully optimized, starting with an initial denaturation at 98°C for 5 min, followed by 25 cycles of denaturation at 98°C for 30 s, annealing at 53°C for 30 s, and extension at 72°C for 45 s. A final extension step at 72°C for 5 min completed the amplification process. To purify the PCR amplicons, Vazyme VAHTSTM DNA clean Beads (Vazyme, Nanjing, China) were utilized, ensuring high-quality amplication products. The purified amplicons were then accurately quantified using the Quant-iT PicoGreen dsDNA Assay Kit (Invitrogen, Carlsbad, CA, USA).

### 2.4 Library preparation and 16S rRNA gene sequence analysis

Subsequently, the amplicons were pooled in equal concentrations and underwent pair-end sequencing with 2*250 bp on the Illumina Miseq platform, utilizing the Miseq Reagent Kit V3 at Shanghai Personal Biotechnology Co.,Ltd (Shanghai, China). To gain insights into the microbiome composition, a comprehensive bioinformatics analysis was conducted using QIIME2 (version 2023.2.0) with adjustments made in accordance with the guidelines outlined in the official tutorials (https://docs.qiime2.org/2023.2/tutorials/). The raw sequence data underwent rigorous processing, including merging, quality filtering, barcode cutting, dereplication, denoising, and chimera removal using the vsearch plugin (v2.22.1, https://github.com/torognes/vsearch). Subsequently, the sequences were clustered into operational taxonomic unit (OTU) and subjected to species taxonomy analysis at a 97% similarity threshold using vsearch. The taxonomic composition of each sample was examined at multiple hierarchical levels, spanning kingdom, phylum, class, order, family, genus, and species. The representative sequence for each OTU was chosen on the sequence with the highest abundance with that OTU. Using the rarefaction approach, the OTU table was normalized to equalize the sequencing depth among samples, aiming for an average depth equivalent to 95% of the lowest sample’s sequence amount. By employing OTU clustering analysis, diversity indices can be utilized to examine the OTUs and evaluate sequencing depth. Statistical analysis of community structure can be performed at various taxonomic data obtained. Additionally, taxonomic classification of each OTU representative sequence was performed by querying the 16S rRNA gene database RDP version 18 (https://mothur.org/wiki/rdp_reference_files) using a confidence threshold of 0.7.

Following this initial analysis, further statistical and visualization examinations of community organization and evolutionary connections may be conducted, including evaluations of alpha diversity. Alpha diversity indices, including observed species, Faith’s phylogenetic diversity (Faith-PD), Chao1 richness estimator, Pielou’s evenness, Shannon diversity index, and Simpson diversity index, were computed using the software usearch v10.0.240 (https://www.drive5.com/usearch/download.html). The statistical significance of variations in alpha diversity among different groups was evaluated using the Wilcoxon rank-sum test. Beta diversity analysis was conducted through principal coordinates analysis (PCoA) to assess the extent of variations in species diversity between sample pairs. Linear discriminant analysis Effect Size (LEfSe) was performed using the website (http://galaxy.biobakery.org/). P-values were calculated using the nonparametric factorial Kruskal-Wallis sum-rank test, within a threshold for the logarithmic liner discriminant analysis (LDA) score of discriminative features set at 2.5. Functional analyses of Metabolic pathways from all domains of life (MetaCyc) pathways was conducted using PICRUSt 2.0 (https://picrust.github.io/picrust/).

### 2.5 Metabolomics analysis

#### 2.5.1 Extraction of compounds

Serum samples were processed under cold conditions, with 100 μL aliquots mixed with 400 μL of methanol/acetonitrile (1:1, v:v) to remove proteins. The resulting mixture underwent centrifugation at 14, 000g for 15 min at 4°. Following centrifugation, the samples were dried in a vacuum centrifuge at 4° to remove excess solvents. Prior to LC-MS analysis, the dried samples were re-dissolved in a solvent consisting of 100 μL acetonitrile/water (1:1; v/v) to ensure optimal conditions for the subsequent analytical steps.

#### 2.5.2 LC-MS/MS analysis

The analysis was conducted utilizing an UHPLC (1290 Infinity LC, Agilent Technologies) tandem with a quadrupole time-of-flight mass spectrometer (AB Sciex TripleTOF 6600) at Shanghai Applied Protein Technology Co., Ltd. For reversed-phase liquid chromatography (RPLC) separation, an ACQUITY UPLC HSS T3 column (1.8 μm, 2.1 mm × 100 mm, Waters, Ireland) was employed. In ESI positive mode, the mobile phase consisted of A - water with 0.1% formic acid, and B - acetonitrile with 0.1% formic acid; while in ESI negative mode, the mobile phase contained A- 0.5 mM ammonium fluoride in water and B - acetonitrile. This comprehensive setup ensured the accurate and reliable separation and detection of the analytes. The column temperature was held constant at 25 °. Each sample was injected in a 2 μL aliquot. The detailed parameters of ionization and acquisition were provided in **Supplementary Table S1**.

#### 2.5.3 Data analysis

The raw MS data underwent a conversion process using Proteo Wizard MSConvert, transforming them into MzXML files. These files were then imported into the freely available XCMS software. For peak picking, we employed parameters such as centWave with a tolerance of 10 ppm, peak width set to range from 10 to 60, and prefilter settings of 10 to 100. Furthermore, we set the peak grouping parameters to bw=5, mzwid=0.025, and minfrac=0.5. To annotate isotopes and adducts, we leveraged the CAMERA (Collection of Algorithms of Metabolite Profile Annotation) tool. During the extraction of ion features, we retained only those variables that possessed more than 50% of nonzero measurement values in at least one group. For the identification of metabolite compounds, we compared the accuracy of m/z values (within 10 ppm) and MS/MS spectra against an internal database comprised of authentic standards. Missing data were filtered using the KNN (K-Nearest Neighbor) method, and extreme values were identified. Finally, the total peak area of the data was normalized to ensure consistency between samples and metabolites. PCA, an unsupervised multivariate statistical method, was conducted on LC-MS data to examine clustering patterns between normal controls and individuals with schizophrenia. PCA was utilized to validate dataset integrity and to visually represent distinctions among sample groups in the unsupervised analysis. In order to differentiate between individuals with schizophrenia and those in the control group, supervised multivariate analysis OPLS were utilized to identify potential metabolites. Metabolites were chosen according to their variable importance in projection (VIP) values, with variables having a VIP >1 significantly influencing the separation of samples in the OPLS-DA analysis. A Student ‘s t-test was conducted to assess the statistical significance of the differential metabolites identified through OPLS-DA modeling across groups (P<0.05). Furthermore, a fold change (FC) threshold was utilized to distinguish between the groups.

### 2.6 Construction of the comprehensive interaction network of gut bacteria and metabolites

The correlation between bacteria and fecal metabolites was assessed using spearman’s rank correlation coefficient based on their relative abundances. The network layout was generated and visualized in a circular format using Cytoscape software (version 3.9.1). Only correlations with a coefficient greater than 0.5 were displayed, and nodes without connection were excluded. Correlations with a coefficient magnitude of 0.5 or higher were chosen for visualization in Cytoscape.

## 3 Results

### 3.1 Clinical profiles of the participant cohort

An in-depth, integrated analysis was conducted on a sample set of 59 individuals, encompassing both schizophrenia and normal control groups, to investigate the bacterial microbiome and fecal metabolome. Detailed participant information was presented in **Supplementary Table S2**, revealing no significant disparities in demographic variables such as gender and body mass index (BMI). We solely focused on the impact of disease on gut microbiota and metabolism, overlooking the potential influence of age on this status.

### 3.2 Gut bacteria differences between schizophrenia and normal control subjects

**Figure 1:**
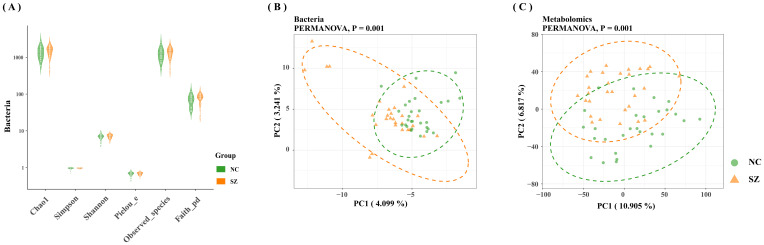
Comparative analysis of gut microbiome characteristics in schizophrenia versus normal controls. **(A)** Depiction of bacterial α-diversity variations between schizophrenia and normal controls. **(B)** Significant differences observed in bacterial signatures between the two groups, as indicated by Bray-Curtis distance and PERMANOVA (P=0.001). **(C)** Distinct metabolic distances exhibited in schizophrenia subjects, significantly differentiated from normal controls based on Bray-Curtis distance and PERMANOVA (P=0.001).

**Figure 2:**
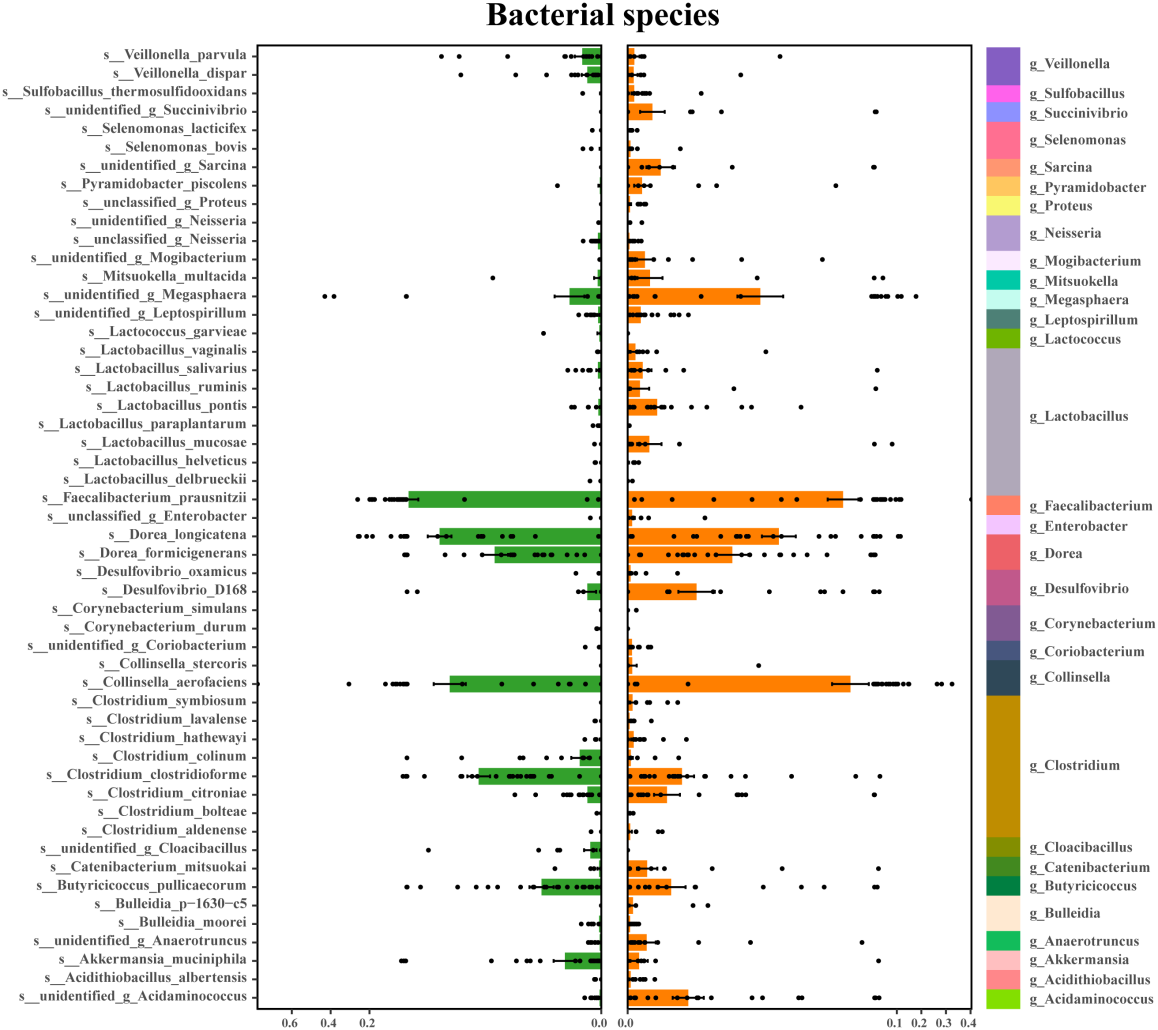
Discriminating bacterial species in schizophrenia and normal controls. Depiction of relative abundances of 30 bacterial species, responsible for distinguishing schizophrenia patients from normal controls. Taxonomic assignments of each species are indicated on the left.

An average of 2,807,456 bases per sample was obtained from the 16S rRNA gene sequencing of 59 fecal samples. Initial analysis of bacterial α-diversity indicated no significant differences among the indexes between the two groups (**Figure 1A**). Subsequently, an investigation was conducted to assess potential disparities in overall bacterial phenotypes between patients with schizophrenia and normal controls, as well as to observe the spatial distribution of the two sample sets. PCoA revealed clear differentiation in bacterial signatures between the two groups based on unweighted Unifrac Distance at the OTU level (**Figure 1B**). To validate these differences statistically and identify biologically significant taxa that differentiate between microhabitats, we conducted LEfSe analysis using a logarithmic LDA score cutoff of ≥ 2.5 and a significance level of P ≤0.05. A total of 30 discriminant bacterial species were identified between the schizophrenia and normal control groups (**Figure 2**; **Supplementary Table S3**). In comparison to normal controls, individuals with schizophrenia exhibited an enrichment of 26 species primarily associated with the genera *Mogibacterium*, *Selenomonas*, *Corynebacterium*, *Coriobacterium*, *Catenibacterium*, *Lactobacillus*, *Acidithiobacillus*, *Sarcina*, *Acidaminococcus*, *Leptospirillum*, *Bulleidia*, *Desulfovibrio*, *Enterobacter*, *Clostridium, Succinivibrio*, *Pyramidobacter*, *Anaerotruncus*, *Proteus*, *Collinsella*, *Cloacibacillus*, *Neisseria*, *Megasphaera*, *Lactococcus*, *Faecalibacterium*, *Dorea and Mitsuokella*. Conversely, the genera *Veillonella*, *Sulfobacillus*, *Butyricicoccus* and *Akkermansia* were found to be down-regulated in individuals with schizophrenia.

### 3.3 Altered fecal metabolites in schizophrenia

**Figure 3:**
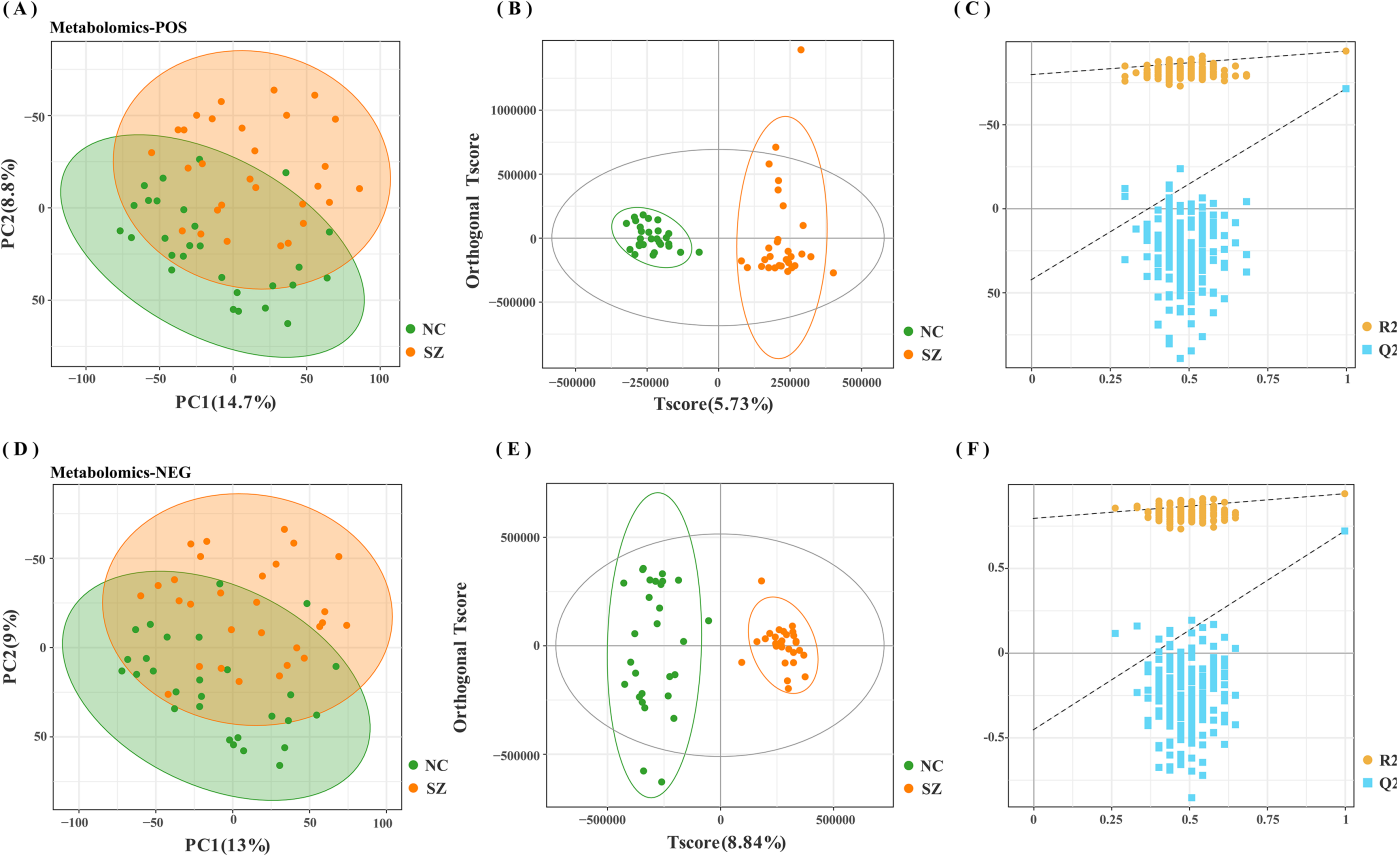
Modeling analysis of schizophrenia and control groups. **(A, D)** Principal component analysis (PCA) representing schizophrenia and control groups in positive and negative modes, respectively. **(B, E)** Orthogonal partial least-squares discriminant analysis (OPLS-DA) models for schizophrenia and control groups in positive and negative modes, respectively **(C, F)** Permutation test analysis of schizophrenia and control groups in positive and negative modes, respectively.

**Figure 4:**
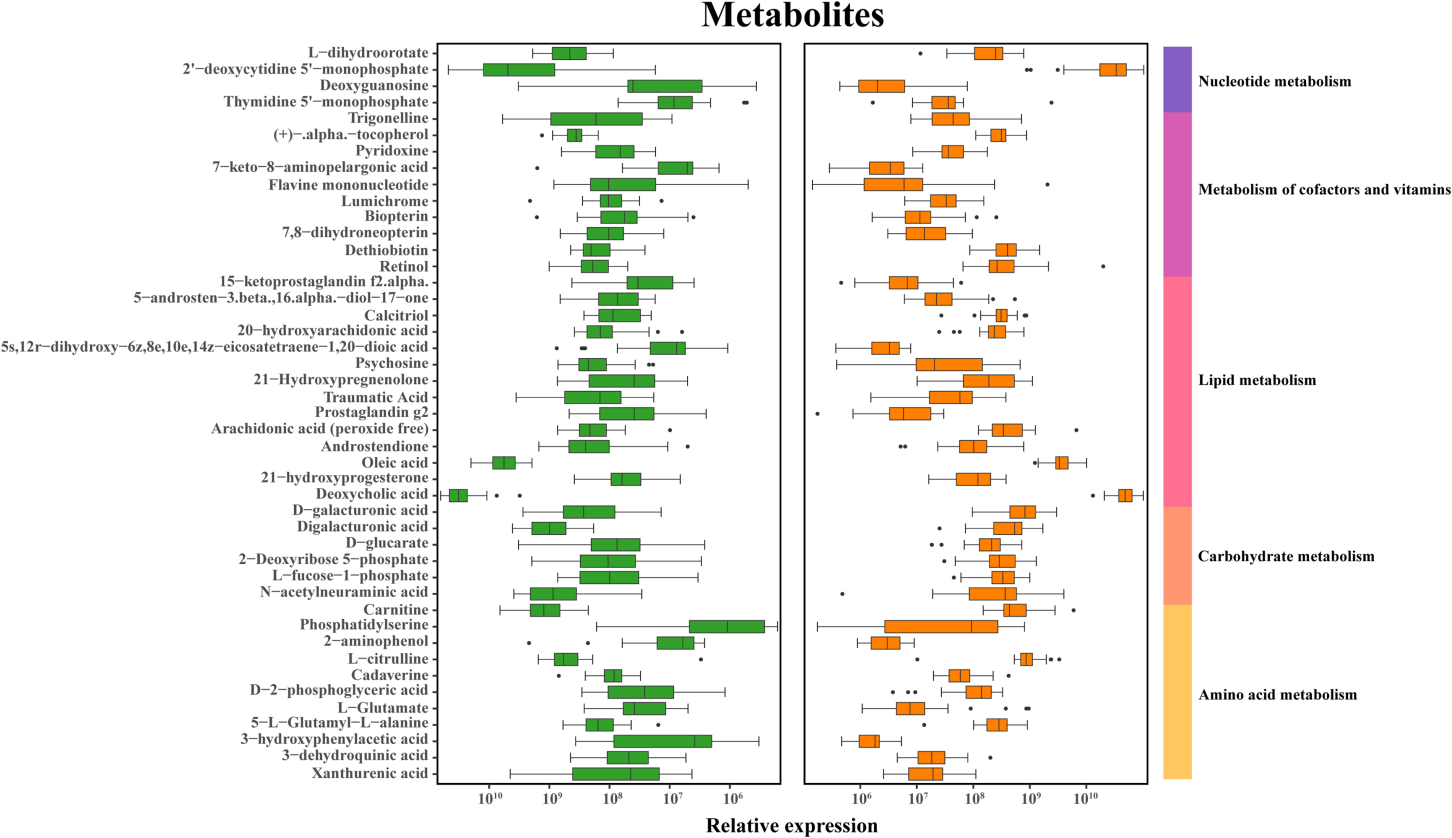
Discriminative fecal metabolites in schizophrenia and normal controls. Depiction of relative abundances of 45 fecal metabolites differentiating between the two groups. These metabolites were mainly involved in carbohydrate, amino acid, energy, lipid and nucleotide metabolism. Identification of discriminative metabolites is based on a LDA score exceeding 1.5.

The fecal metabolic markers identified through metabolomics were presented in **Figure 1C** and **Supplementary Table S4**. The processed and standardized datasets acquired through LC-MS were utilized to construct PCA models for visualizing the clustering of samples (**Figures 3A, D**). The PCA scores clearly indicated a distinct segregation between the schizophrenia and normal control cohorts, as evidenced by the parameters PC_1 =_ 14.7% and PC_2 =_ 8.8% for the positive mode, and PC_1 =_ 13% and PC_2 =_ 9% for the negative mode. These results underscored the efficacy of our modeling approach and the thoroughness of our data interpretation. To further identify the key contributors to this differentiation, an OPLS-DA was conducted, with a criterion of VIP >1, as depicted in **Figures 3B**, **E**. The assessment of the OPLS-DA model’s quality was conducted through the examination of R2Y and Q2 parameters, including the model’s fitness and predictive capabilities. A threshold value of R^2^ greater than 0.4 and a truncation value of Q^2^ in the Y-axis less than 0 were deemed reliable for the establishment and validation of clinical models (**Figures 3C, F**). Furthermore, significant differences were observed in the global metabolic spectrum between individuals with schizophrenia and normal controls. The most significantly expressed metabolites depicted in **Figure 4** were determined through the application of LEfSe analysis with a threshold of 1.5, and their specific details were documented in **Supplementary Table S5**. In comparison to the control group, individuals with schizophrenia exhibited a dysregulation in 45 metabolites, primarily associated with amino acid metabolism (including cystine, citrulline, 1-methyl-l-histidine, 3,4-dihydroxy-l-phenylalanine, 3-hydroxykynurenine and 5-hydroxy-l-tryptophan), as well as nucleotide metabolism, fatty acid metabolism, carbohydrate metabolism, and glycerophospholipid metabolism.

### 3.4 Co-occurrence analysis of bacteria and metabolites

**Figure 5:**
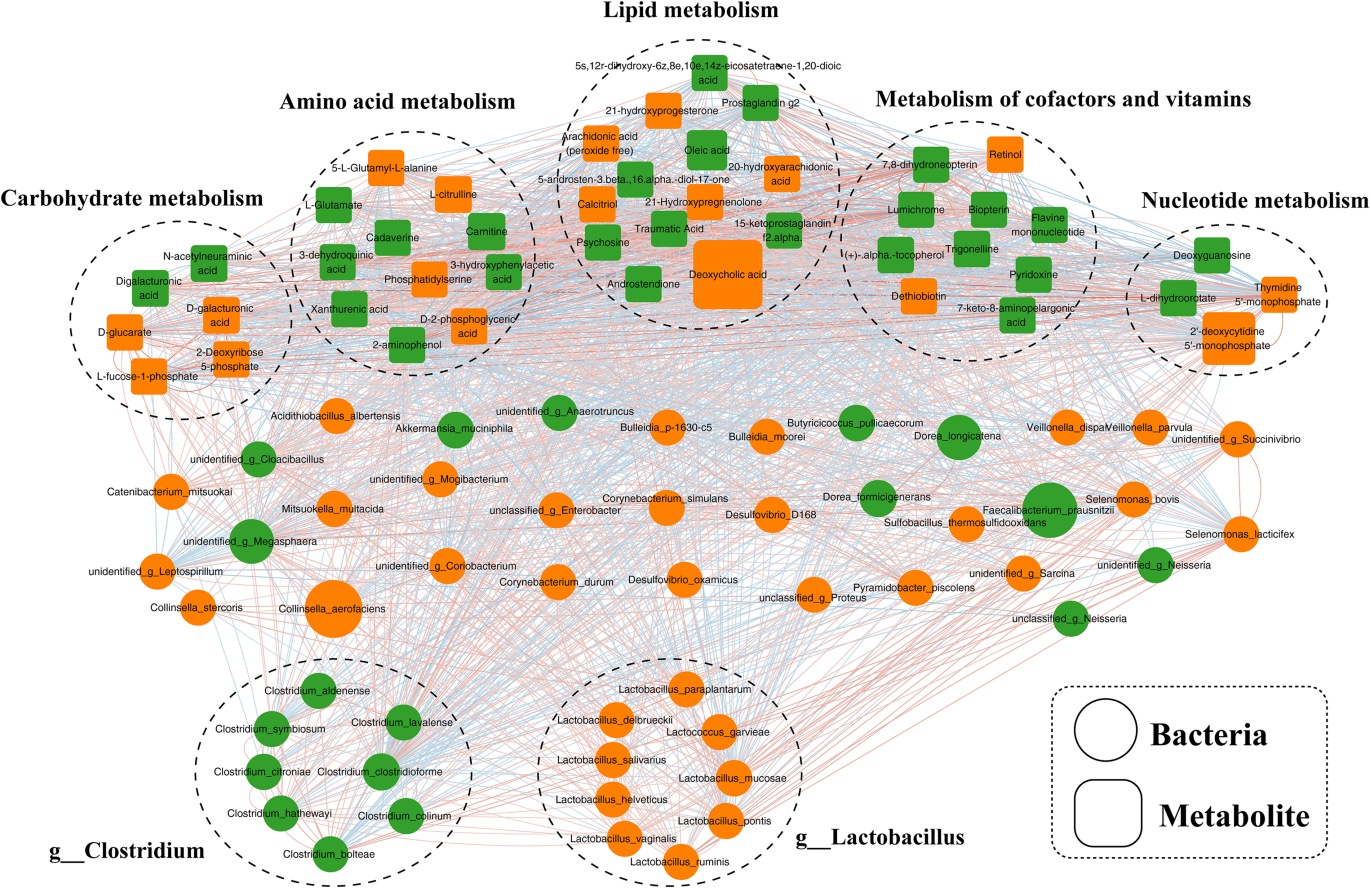
Co-occurrence network of differential bacterial species and fecal metabolites in schizophrenia. A network constructed from the relative abundances of bacterial species and fecal metabolites exhibiting differential patterns in schizophrenia subjects, representing co-occurrence relationships.

An investigation into the potential relationships between the abundances of distinct gut microbiome and fecal metabolites was conducted to elucidate the underlying regulatory network. Overall, co-occurrence analysis revealed robust and extensive associations between bacterial species and fecal metabolites, as illustrated in **Figure 5**, the corresponding associated parameters were provided in **Supplementary Table S6**. In this co-expression network, the bacterial species involved were predominantly categorized into 2 clusters, with cluster 1 consisting of 8 enriched bacterial species from the genus *Clostridium* and cluster 2 predominantly including 9 species from the genus *Lactobacillus*, with additional species assigned to *Collinsella* and *Selenomonas*. Our analysis revealed a lack of correlation between cluster 1 and cluster 2. Furthermore, the members of these clusters exhibited varying degrees of positive or negative correlations with the metabolites.

### 3.5 Variations in microbial functionality and fecal metabolites in schizophrenia

**Figure 6:**
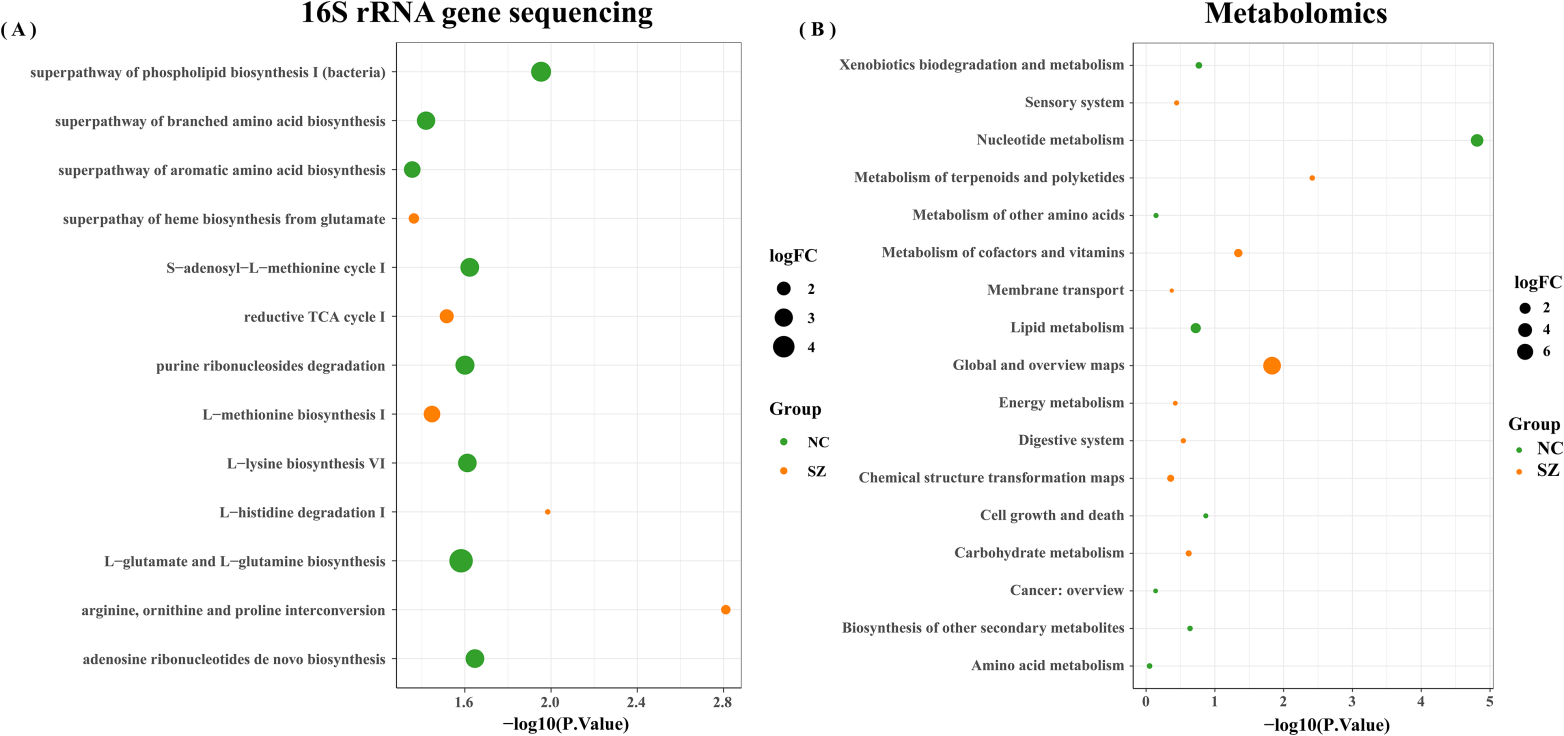
Enrichment analysis. Enrichment analysis of biological processes linked to differential microbial genes **(A)** and metabolites **(B)** in schizophrenia patients and normal controls.

A total of 13 differential MataCyc pathway were identified between the schizophrenia and control groups. These differential pathways were predominantly associated with 13 biological processes (**Figure 6A**), with a primary focus on metabolic pathways including superpathway of branched amino acids biosynthesis; arginine, ornithine and proline interconversion; L-glutamate and L-glutamine biosynthesis; histidine degradation; methionine biosynthesis; superpathway of aromatic amino acid biosynthesis; superpathway of phospholipid biosynthesis and TCA cycle. Meanwhile, metabolomics analysis showed that pathway enrichment was particularly involved in amino acid metabolism, lipid metabolism, and nucleotide metabolism (**Figure 6B**). Integration analysis of these enriched metabolic pathways indicated that disturbances in amino acid metabolism were particularly relevant to the gut ecosystem in schizophrenia.

**Figure 7:**
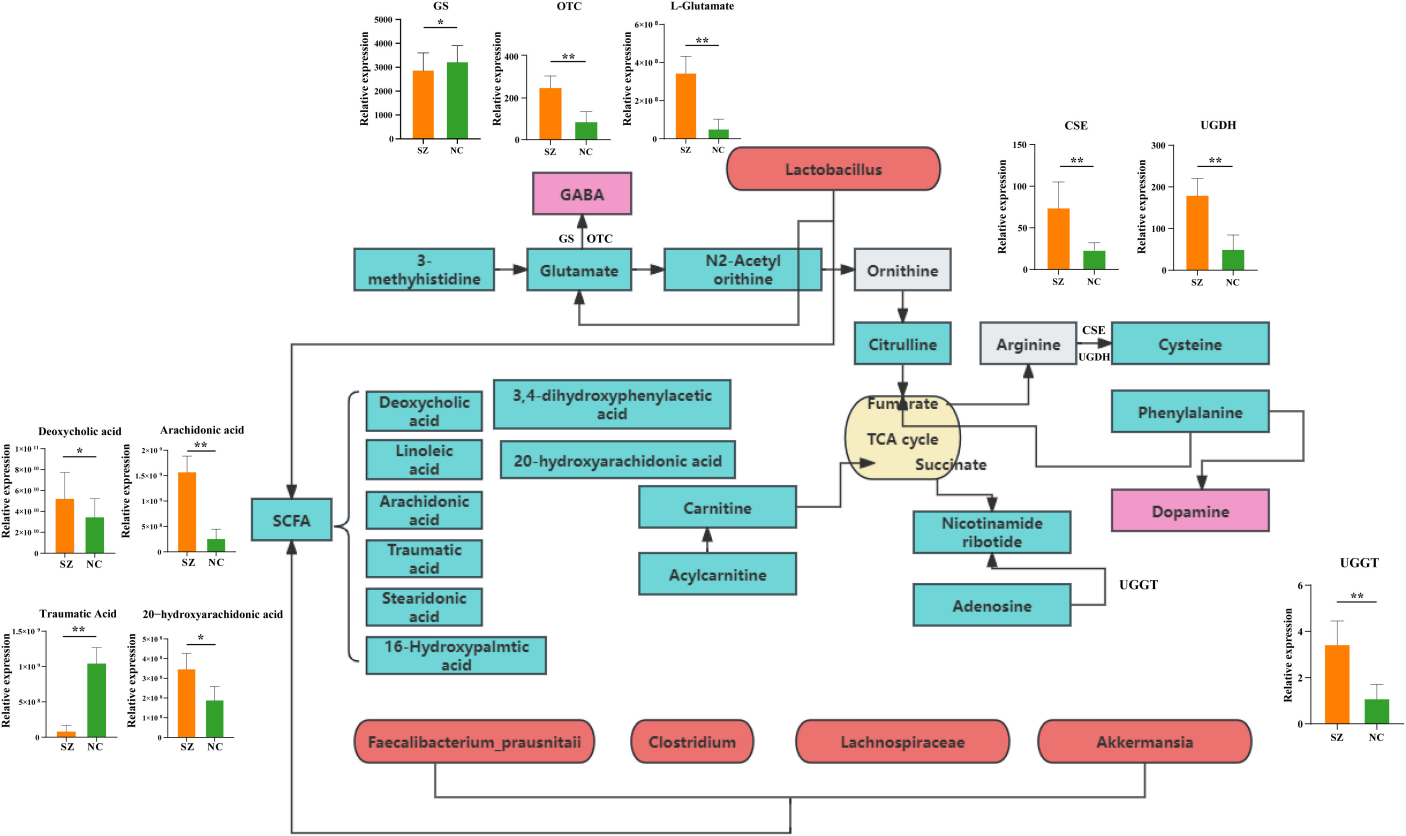
Mapping of key metabolic pathways in the gut ecosystem via microbial genes and fecal metabolism. Illustration of metabolic pathways identified through the analysis of microbial genes and fecal metabolites withing the gut ecosystem. GS, Glutamine synthetase; OTC, Ornithine transcarbamylase; CSE, Cystathionine gamma-lyase; UGDH, UDP-glucose 6-dehydrogenase; UGGT, UDP-glucose glucosyltransferase.

The PICRUSt 2.0 was used to investigate the specific contributions of the gut microbiome to the regulation of fecal amino acid metabolism through the identification of various enzyme commission numbers (ECs) associated with disturbed amino acid metabolism. A total of 379 differential ECs were identified in patients with schizophrenia compared to normal controls. These microbial genes and fecal metabolites were found to be concurrently associated with pathways related to histidine, glutamate, and GABA metabolism, as well as phenylalanine and dopamine. Specifically, elevated levels of glutamate were observed in the fecal samples of individuals with schizophrenia compared to the control group (**Figure 7**). Consistent with this observation, dysregulation of microbial enzyme-related genes (GS, OTC) was also detected in the schizophrenia group. These findings suggest that the metabolism of glutamate to GABA in individuals with schizophrenia may be influenced by a variety of microbial species, potentially contributing to the pathogenesis of schizophrenia. Additionally, our study revealed elevated expression of genes (CSE and UGDH) involved in arginine catabolic pathways in the schizophrenia group compared to the control group, indicating enhanced fecal arginine degradation in individuals with schizophrenia. Furthermore, the increased levels of the downstream catabolic product of cysteine in the schizophrenia group support this observation. Our observations indicated an enrichment of the gene (UGGT) in individuals with schizophrenia compared to those in the control group, potentially leading to the activation of adenosine metabolism. Moreover, variations in fatty acids involved in lipid metabolism were noted.

## 4 Discussion

Utilizing an untargeted UPLC-MS platform and 16S rRNA gene sequencing, the study characterized the composition and interactions of distinct bacterial species and fecal metabolites in a cohort of 29 schizophrenia patients and 30 normal controls. PCA and OPLS-DA scores were employed for multivariate statistical analysis of the metabolites. The findings from both PCA and OPLS-DA analysis indicated successful differentiation between the schizophrenia and normal control groups. Our observation revealed significant differences in fecal metabolites between schizophrenia patients and the control population. Additionally, α-diversity and β-diversity analyses demonstrated varying abundances of gut microbiota between the two groups. The enrichment pathways of metabolites aligned with gut microbiota, particularly emphasizing amino acid and lipid metabolism. The disruption of amino acid metabolism in the gut ecosystem of individuals with schizophrenia represents a notable characteristic. However, we should also need to note the fact that the schizophrenia patients involved in this study are long term inpatients and have undergone long-term systematic medication, which will have a certain impact on the patients’ metabolism and intestinal microorganisms. Since this study focuses more on the relationship between the occurrence and development of schizophrenia and the body’s metabolism, the administration of antipsychotic drugs has not been included as an influencing factor. This study adopts 16S gene sequencing technology, which does not identify species to the species level, so there are defects om species identification. For the pathway analysis of this technology, the predictive function PICRUSt analysis is used, which may not fully capture the actual functions of microbial communities. In the future experiments, we will reveal the conditions of schizophrenia, organismal metabolism and intestinal microorganisms from the longitudinal dimension based on metagenomics.

Numerous studies have delved into the intricate relationship between schizophrenia and intestinal microbiota, substantiating their multifaceted connection with the onset and progression of schizophrenia (31, 32). Our research contributes to understanding the potential contributions of the gut ecosystem to the pathogenesis of schizophrenia and supports the development of objective diagnostic approaches for this disorder.

Prior investigations into the relationship between gut microbiota and central nervous system disorders have primarily concentrated on disease profiling or in-depth analyses of disease onset and progression (33, 34). Using the 16S rRNA gene sequencing method, one study identified an enriched presence of phylum *Firmicutes*, *Bacteroidetes*, *Planctomycetes*, and *Actinobacteria* in individuals with schizophrenia compared to those with schizophrenia as well as those without metabolic syndrome (35). Additionally, our analysis revealed a distinct bacterial composition in individuals with schizophrenia compared to normal controls, with 30 bacterial species specifically associated with the occurrence of schizophrenia. This step is essential and serves as a prerequisite for identifying the key species linked to the development and advancement of schizophrenia. Our research revealed that the enriched species distinguishing individuals with schizophrenia primarily belonged to the genera *Clostridium* and *Lactobacillus*. *Clostridium* plays a crucial role in the interactions between gut microbiota and the host, particularly in metabolic pathways and inflammation (34). Additionally, the increased presence of *Lactobacillus* and *Desulfovibrio* is associated with the severity of autism (36). Furthermore, it is suggested that *Lactobacillus* may play a role in the pathophysiology of schizophrenia by interacting with GABAergic and glutamatergic neurotransmission (34). This is supported by the significant correlation between cluster 1 of *Clostridium* and cluster 2 of *Lactobacillus* with amino acid and lipid metabolism. Additionally, a decrease in the *Akkermansia* genus was observed in the schizophrenia group compared to the normal control group. *Akkermansia muciniphila* has been shown to have beneficial effects on host health by enhancing immunological and metabolic functions (37).

The current consensus in the scientific community is that gut bacteria play a crucial role in influencing various metabolic pathways in the host. This study further validates this concept through occurrence analysis. The co-expression network analysis revealed a strong correlation between altered bacterial species, particularly those belonging to the *Clostridium* and *Lactobacillus* genera, and fecal metabolites involved in amino acid and lipid metabolism. Importantly, all identified differential microbial species, and fecal metabolites were consistently associated with amino acid metabolism. Disruptions in the levels of 5-hydroxytryptamine, glutamate, and dopamine within the gastrointestinal tract, peripheral blood, and central nervous system have been identified as significant factors in the pathogenesis of schizophrenia through the brain-gut axis (38–40). Recent research has suggested that alterations in the gut microbiome or specific microbial species may serve as indicators for the development of mental disorders by influencing the levels of amino acid neurotransmitters, including short-chain fatty acids (41), tryptophan metabolism (42), and gamma-aminobutyric acid (43). This study revealed dysregulation of fecal glutamate levels and its associated metabolites (citrulline, glutamate, and 3-methylhistidine) in individuals with schizophrenia compared to normal controls. Histidine, an essential proteogenic amino acids with anti-inflammatory and antioxidant properties, demonstrated potential for mitigating kidney damage and enhancing clinical treatment strategies for disease management (44). In alignment with the findings of this investigation, a decrease in histidine levels was similarly noted in females with obesity syndrome (45). Our observations revealed that the concentrations of histidine and its associated metabolites in individuals with schizophrenia were notably diminished compared to those in non-afflicted individuals, suggesting that the depletion of histidine in schizophrenia patients may impede their capacity for reactive oxygen species clearance and anti-inflammatory responses, consequently impacting the onset and progression of the disorder.

Glutamate, a key component in the central nervous system, is involved in the enteric nervous system and the cerebral intestinal axis, thus playing a significant role in this pathway (46). The glutamate neurotransmitter is presented in the intricate network of the enteric nervous system and contributes to the local regulation of gastrointestinal function. Research indicates that glutamate is essential for the activation of the gut-brain axis and the maintenance of energy balance. Glutamate activation of vagus afferent fibers occurs through the stimulation of glutamate receptors and cell transduction molecules in intestinal epithelial cells, subsequently targeting the central nervous system (47). The inter play between glutamate and the GABA system is a key factor in the pathophysiology of schizophrenia. Dysfunction of NMDA receptors located on cortical GABA interneurons could potentially lead to insufficient inhibition of glutamatergic projections within the brain, leading to excessive stimulation of intermediate dopamine neurons (48). Alterations in glutamate metabolism intermediates provide additional evidence of the connection between neurotransmitters associated with this pathway and schizophrenia. Consistent with these finding, two microbial enzyme-related gene involved in the conversion of glutamate to GABA found to be up-regulated in the schizophrenia cohort. The precise mechanisms by which cystine influences schizophrenia remain incompletely elucidated, the evidence suggest that cystine’s interaction with glutamatergic synaptic transmission may underlie the behavioral manifestations associated with schizophrenia or related disorders. Elevated levels of cystine appear to enhance glutamate transport activity, leading to hyperactivation of NMDA receptors and subsequent degenerative changes and apoptosis of neuronal cells, ultimately contributing to the symptomatology of schizophrenia (49). Consistent with these results, two enzymes involved in this pathway also showed an increase in the schizophrenia group.

Furthermore, our study revealed significant upregulation of metabolites linked to nicotinamide metabolism, including as adenosine and nicotinamide, in individuals with schizophrenia, suggesting an activation of fecal adenosine degradation. Meanwhile, we found that a microbial enzyme-related gene involved in the metabolic pathway also increased in schizophrenia group. Inosine and adenosine have demonstrated the ability to promote synaptic reconnection, indicating that adenosine may emerge as a promising candidate for therapeutic target for conditions such as spinal cord injury and stroke (50).

The study examined metabolites related to fatty acids, specifically focusing on the pathway associated with phospholipid metabolism. Microbial activity in the gastrointestinal tract facilitates the breakdown of dietary proteins and carbohydrates into various fatty acid, including linoleic acid, arachidonic acid, deoxycholic acid and traumatic acid. These short-chain fatty acids serve crucial physiological roles within the gut, including supplying energy to colon cells, modulating the intestinal immune response, and promoting intestinal well-being (51, 52). Fatty acids exhibit antibacterial properties that are influenced by variables including carbon chain length, saturation and double bond placement (53). Our study revealed a significant decrease in the levels of traumatic acid in schizophrenia patients compared to normal controls, suggesting a disruption in fatty acid metabolism. Traumatic acid, a potent wound-healing agent in plants, was also detected in the urine of children with asthma (54). Additionally, linoleic acid and its derivatives, such as arachidonic acid, are not only influenced by dietary intake but also play a crucial role in inflammatory response with an upregulation trend (55). The dysregulation of lipid metabolism in schizophrenia is a significant aspect of the disease, potentially contributing to the development of cardiovascular disease and metabolic syndrome (56). The fatty acids studied in this research exhibited correlations, both positive or negative, with the *Clostridium*, *Lactobacillus*, *Selenomonas*, and *Desulfovibrio* genera, indicating a complex interplay within the gut microbiome. Assessing the impact of gut microbiota on the host and the production of metabolites through microbiome fermentation on intestinal substrate is a complex regulatory process, with both positive and negative implications.

## 5 Conclusion

In this study, a combined approach utilizing LC-MS based metabolomics and 16S rRNA gene sequencing was employed to delve into the metabolic disturbances and gut microbiota associated with schizophrenia, aiming to elucidate the intricate metabolic network involved in this disorder. The dysregulation of amino acid and sphingolipid metabolism has been implicated in the pathophysiology of schizophrenia, potentially linking gastrointestinal and neurological functions. The utilization of metabolomics and innovative analytical techniques not only elucidates these metabolic abnormalities but also sets the stage for further research endeavors focused on enhancing early detection, accurate diagnosis, and a comprehensive comprehension of the pathophysiological pathways associated with schizophrenia. Indeed, the study is subject to limitations stemming from the relatively small sample size, in the following research, we will broaden our sample size to ensure the feasibility of the results.

## Data Availability

The data presented in the study are deposited in the NCBI Sequence Read Archive repository, accession number PRJNA1077648.
