# Supplementary material for: Unveiling the gut microbiota blueprint of schizophrenia: a multilevel omics approach
Source: Front Psychiatry. 2024 Sep 25;15:1452604. doi: 10.3389/fpsyt.2024.1452604 (PMC11461293; doi:10.3389/fpsyt.2024.1452604)
Supplement: Supplementary file 1 [file Presentation1.pdf]

Supplementary Material

Unveiling the gut microbiota blueprint of schizophrenia: A multilevel omics approach

DongDong Qi[1†], Peng Liu[1†], YiMeng Wang[2], XuGuang Tai[1], ShiFa Ma[1*]

***Correspondence**
ShiFa Ma
m13947060575_2@163.com

† These authors contributed equally to this article.

Supplementary Table Captions

Table S1. Detailed elution and detection parameters of liquid chromatography- mass spectrometry.
Table S2. A comprehensive overview of the clinical characteristics exhibited by the enrolled participants in this study.
Table S3. Identification of distinct bacteria species that differentiate schizophrenia patients from normal control groups.
Table S4. Identification of distinct fecal metabolites that distinguish schizophrenia patients from normal control groups.
Table S5. Details of 45 differentially expressed fecal metabolites with an LDA score > 1.5.
Table S6. The person correlation analysis of microbiota and metabolites.
