# Supplementary material for: Unveiling the gut microbiota blueprint of schizophrenia: a multilevel omics approach
Source: Front Psychiatry. 2024 Sep 25;15:1452604. doi: 10.3389/fpsyt.2024.1452604 (PMC11461293; doi:10.3389/fpsyt.2024.1452604)
Supplement: Supplementary file 2 [file Table1.docx]

| **LC elution procedure** | | **MS condition** | **Parameters** |
| --- | --- | --- | --- |
| Retention time | B% | Ion source gas 1/2 | 60 psi |
| 1.5 | 1 | Curtain gas | 30 psi |
| 11.5 | 99 | Temperature | 600℃ |
| 11.6 | 1 | Declustering potential (positive/negative) | 60 V/−60V |
| 15 | 1 | Ion spray voltagefloating (positive/negative) | 5500V/−5500V |
|  |  | Accumulation time | 40 ms |
|  |  | Collision energy (positive/negative) | 30V/−30V |
|  |  | Collision energy spread | 35V |
|  |  | m/z range | 25-1000 Da |
|  |  | Scan accumulation time | 0.05 s/spectra |
