# Supplementary material for: Unveiling the gut microbiota blueprint of schizophrenia: a multilevel omics approach
Source: Front Psychiatry. 2024 Sep 25;15:1452604. doi: 10.3389/fpsyt.2024.1452604 (PMC11461293; doi:10.3389/fpsyt.2024.1452604)
Supplement: Supplementary file 3 [file Table2.docx]

| **Parameters** | **Metabolomics/16S rRNA gene sequence analysis** | | **P-value** |
| --- | --- | --- | --- |
|  | **Schizophrenia** | **Normal Controls** | **-** |
| Number(n) | 29 | 30 | - |
| Gender(female/male) | 14/15 | 15/15 | 4.22e-01 |
| Age (in years) | 46.28±9.49 | 30.87±5.41 | 1.38e-07 |
| BMI | 25.20±4.22 | 24.35±3.26 | 3.91e-01 |
| Nation（Han/Mongolia） | 27/2 | 21/9 | - |
| Subdivision disorder (Paranoid/undifferentiated/unknown subtype) | 16/11/2 | - | - |
